# Strategies to Protect Dialysis Patients against Bisphenol A

**DOI:** 10.3390/biom11091375

**Published:** 2021-09-17

**Authors:** Borja Quiroga

**Affiliations:** Nephrology Department, Hospital Universitario de la Princesa, 28806 Madrid, Spain; borjaqg@gmail.com; Tel.: +34-915202200

**Keywords:** bisphenol A, endocrine disruptor, haemodialysis

## Abstract

Bisphenol A (BPA), also known as 2,2,-bis(4-hydroxyphenyl) propane, is a common component of plastics worldwide. However, it has been shown to act as an endocrine disruptor with some hormonal functions. Furthermore, high levels of BPA have been related to the development of cardiovascular events and the activation of carcinogenesis pathways. Patients with chronic kidney disease (CKD) have higher serum concentrations of BPA due to their impaired renal function. This situation is aggravated in CKD patients requiring dialysis, because the BPA content of dialysis devices (such as, for example, the filters) is added to the lack of excretion. In addition to the development of BPA-free dialysis filters, some techniques can contribute to the reduction of BPA levels in these patients. The aim of this review is to illustrate the impact of BPA on dialysis patients and suggest some strategies to reduce its inherent risks.

## 1. Introduction

**Figure 1:**
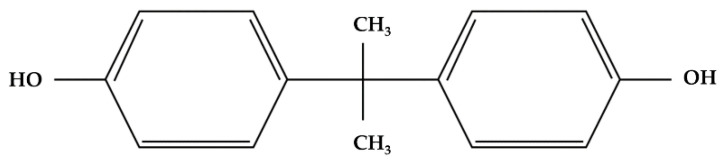
Structure of bisphenol A (BPA).

Bisphenol A (BPA), also known as 2,2,-bis(4-hydroxyphenyl) propane, is a common component of synthetic polycarbonate plastics and epoxy resins (Figure 1).

BPA has been used extensively worldwide in a variety of products, including drink and food containers, glasses, compact discs, electronic devices, and even baby toys or bottles [1]. Medical devices are not an exception, and many of the materials used daily in clinical practice have different concentrations of BPA [2]. Based on urinary analysis, we currently know that more than 90% of the population (especially in developed countries) are exposed to BPA [3,4].

Despite its indiscriminate use, BPA is now considered an endocrine disruptor that is able to alter several signalling pathways [5]. Its potential effects include thyroid and hepatic dysfunction, obesity, inflammation, reproductive toxicity, and even carcinogenesis induction or cardiovascular event promotion (Table 1) [6,7].

International campaigns have been conducted in order to reduce or eliminate the BPA content in commonly used products, especially those used by babies or children. The initiative “BPA-free” established a special label to inform customers about the concentration of BPA in plastic containers. Concern about the accumulation of BPA has contributed to the implementation of devices in water treatment plants to reduce its levels [8]. 

The plastics industry has sought alternatives for avoiding the extensive use of BPA, such as the inclusion of BPA analogues (bisphenol S, bisphenol F, and bisphenol B) in the manufacture of different devices. Although, in theory, BPA analogues present a safer profile, some recent studies have demonstrated important toxic events related to their use [9,10]. However, the real toxicological impacts of bisphenol analogues need to be addressed with additional studies [11]. 

The aim of the present review is to summarise the evidence of kidney-induced BPA injury and the potential for attenuating the accumulation of this compound in dialysis patients.

## 2. Accumulation of Bisphenol A in Patients with Chronic Kidney Disease

After ingestion, BPA is metabolized in the liver via glucuronidation and sulfation pathways (half-life: 5.3 h). BPA metabolites are mainly excreted by urine; thus, kidney dysfunction raises their blood levels. Of note, BPA binds with serum albumin up to 75%; this consideration is highly important when analysing its reduction with extra renal techniques [12]. In population-based studies, BPA levels have been shown to be inversely correlated to kidney function, with maximum levels observed in dialysis-dependent patients [13]. In this regard, a recent study including data of the US National Health and Nutrition Examination Survey (NHANES) 2005–2016 suggested that exposure to BPA may be responsible for impaired kidney function (low glomerular filtration rate (GFR) and increased albuminuria) [14]. In addition to their impaired renal excretion, haemodialysis and peritoneal dialysis (PD) patients are exposed to medical devices with variable concentrations of BPA. In this regard, as shown by Shen et al., haemodialysis filters present different concentrations of BPA, which depend on the membrane composition and are higher in polysulfone membranes [15]. In order to avoid the deleterious effects of BPA, a BPA-free filter made of polynephron is now available [16]. Evidence in peritoneal dialysis is limited. The few published papers regarding this procedure have shown that PD patients have lower BPA levels in comparison to haemodialysis patients. Nevertheless, some sources of concern should be addressed in this population, such as the influence of residual renal function (more frequent in PD than in haemodialysis patients) or the influence of the different techniques on BPA concentrations [9,17]. 

## 3. Bisphenol A-Linked Vascular and Kidney Damage

One of the first declared effects of BPA was hypertension. A randomized crossover clinical trial conducted in elderly adults demonstrated a strong association between drinking canned beverages and blood pressure elevation two hours after intake [18]. This effect, previously suggested in large epidemiological studies, was thereby confirmed in this trial [19]. Several mechanisms have been implicated in regard to BPA-induced hypertension, with most of them related to direct endothelial damage caused by BPA. Experimental studies in mice showed the capacity of BPA to induce hypertension and upregulate angiotensin II and calcium-calmodulin kinase II in murine endothelial cells, inhibiting the production of nitric oxide and promoting oxygen free radicals. Taken together, all these effects can be considered to be responsible not only for elevating blood pressure, but also for vascular damage [20]. 

Linked to vascular damage, BPA is especially toxic for some kidney cells. From a clinical point of view, a recent study conducted by Nie et al. showed that higher BPA levels were significantly associated with the incidence of chronic kidney disease (CKD) [21]. Beyond the unquestionable role of hypertension in the development of CKD, BPA has direct toxic effects on podocytes, one of the components of the glomerular filtration barrier. In this regard, Olea-Herrero et al. demonstrated that low and high BPA concentrations were able to induce in vitro and in vivo podocyte injury, promoting their hypertrophy, apoptosis, and subsequent podocytopenia [22]. Consequently, podocyte damage leads to the development of proteinuria, a well-known risk factor for the incidence and progression of CKD [23]. 

Interestingly, a recent study performed by Ruiz-Priego et al. showed that BPA could deregulate autophagy and promotes inflammatory infiltration and tubular and renal fibrosis, showing new BPA-mediated damage pathways [24]. 

A recent meta-analysis conducted by Moreno-Gomez-Toledano et al. summarised all of the published evidence regarding the effects of exposure to BPA on kidney diseases, highlighting its strong association with the incidence of CKD, albuminuria, and glomerular filtration rate [25].

## 4. Reducing Bisphenol A in Haemodialysis Patients

Patients on dialysis are at high risk of developing cardiovascular events, a situation that can be aggravated by BPA. For this reason, it is desirable to reduce BPA levels in this population, especially in patients with comorbidities or in low-income countries where the reuse of filters is still common in clinical practice. 

### 4.1. Changing the Filter

The accumulation of BPA in haemodialysis patients is a consequence of kidney impairment and the use of medical devices with BPA-containing plastics, especially filters. It is important to emphasise that not all filters are manufactured with BPA. For example, polysulfone and polyamide membranes show the highest amounts of BPA [15]. In contrast, polynephron filters are BPA-free dialyzers and, as demonstrated by Mas et al. in a prospective study, their use contributes to reducing BPA levels in dialysis patients [16]. Interestingly, a previous report by the same group demonstrated that the use of a BPA-free membrane (polynephron) decreased BPA concentrations in peripheral blood mononuclear cells in comparison to the use of a polysulfone membrane [26]. In this regard, it is unknown how these BPA-free filters could affect other endocrine disruptors (such as perfluorochemicals) [27].

### 4.2. Changing the Technique 

Haemodialysis techniques have progressed in the last 15 years, improving patients’ survival, as well as their rate of cardiovascular events and quality of life. One of the most relevant advances was the addition of convective solute transport to the diffusive mechanism, which provides a greater reduction of middle-sized molecules (< 50 kDa). The accumulation of those middle-sized molecules, also known as uremic toxins, has been related to worse outcomes, whereas their removal has been associated with important improvements in haemodialysis prognosis [28]. Considering this background, our group designed a paired prospective study to evaluate the usefulness of adding high-volume convective transport (online hemodiafiltration (OLHDF)) to the usual haemodialysis techniques (high-flux haemodialysis (HF-HD)) in order to reduce BPA. We were able to demonstrate that OLHDF reduced BPA levels after 3 weeks, in contrast to HF-HD. Moreover, when patients switched from OLHDL to HF-HD, BPA levels rose again [29]. Table 2 shows the BPA levels of haemodialysis patients in comparison to the general population.

### 4.3. Changing the Plastic 

Finally, the use of BPA analogues, such a BPS, has emerged as an alternative. To date, no dialysis filters have been built using only BPS. However, a recent study by Mas et al. demonstrated that BPS concentrations were 10-fold higher in dialysis patients than in healthy subjects. Interestingly, the exposure of cultured tubular cells to BPS showed no biological effect in terms of cytotoxicity, inflammation, or oxidative stress, thus presenting BPS as an interesting alternative [30]. 

Although the toxic effect of BPA burden in dialysis patients remains controversial, the mere possibility of enhancing oxidative stress and inflammation in these patients should call the attention of nephrologists. 

## 5. Conclusions

BPA acts as an endocrine disruptor and can enhance cardiovascular risk. In patients with CKD, especially those on dialysis, BPA levels are elevated due to renal impairment. Nephrologists should apply strategies to reduce BPA exposure in order to avoid its deleterious effects.

## Figures and Tables

**Table 1:** BPA potential effects.

Reproductive dysregulation: sexual dysfunction and reduction of semen quality
Reduction of testosterone secretion
Obesity
Diabetes and insulin resistance
Albuminuria
Incidence of CKD
Hypertension
Thyroid nodules and thyroid dysfunction (▲thyroxine and ▼TSH)
Hepatic toxicity
Cancer
Cardiovascular disease
Behavioural disorders
Epigenetic modifications (DNA methylation)
Pro-inflammatory and pro-oxidant
Pregnancy outcomes: preeclampsia, prematurity, pregnancy loss

Abbreviations: CKD: chronic kidney disease, TSH: thyroid-stimulating hormone.

**Table 2:** BPA serum levels in the general population and in haemodialysis patients (with the different techniques).

	General Population	Haemodialysis Patients	
		HF-HD	OL-HDF
BPA levels (ng/mL)	3.25 (0.59–14.89)	7.5 ± 3.5	6.7 ± 2.5

Abbreviations: HF-HD: high-flux haemodialysis; OL-HDF: online hemodiafiltration. Data refer to total BPA levels.
